# Twelve-step Approach for Academic Development Planning in Academic Surgery: Climbing the Academic Uphill With Strong and Steady Steps

**DOI:** 10.1097/AS9.0000000000000119

**Published:** 2022-01-06

**Authors:** Sruthi Selvakumar, Darwin Ang, Adel Elkbuli

**Affiliations:** From the * NSU NOVA Southeastern University, Dr. Kiran C. Patel College of Allopathic Medicine, Fort Lauderdale, FL; † University of Central Florida, Ocala, FL; ‡ Department of Surgery, Ocala Regional Medical Center, Ocala, FL; § University of South Floria, Tampa, FL; ∥ Department of Surgery, Division of Trauma and Surgical Critical Care, Orlando Regional Medical Center, Orlando, FL.

**Keywords:** academic development, academic planning, surgical mentoring

## Abstract

Mini-abstract: Surgical trainees and young academic surgeons should understand the specifics of the career they are pursing, and surgeon mentors should enhance their leadership roles and support the recruitment and retainment of academic surgeons through effective academic career support and strategic planning. Utilizing academic development planning can serve as an essential tool as both mentors & mentees navigate different career choices towards achieving a successful and sustainable academic career advancement.

## INTRODUCTION

Academic surgery is a critical aspect of medical education and our healthcare system; however, the field is dogged by staff shortages, barriers to entry, and high rates of burnout (30%–38%).^1^ To address shortages and barriers to entry, we should cultivate junior faculty, resident/fellow, and medical student interest in academic surgery and provide them with resources and support to prepare for the rigor of academic surgery. A key aspect of this could be an academic development plan (ADP) for those interested in the field, tailored to address the unique demands of academic surgery. An increased focus on preparation for an academic career early on could foster enthusiasm toward academia among rising physicians and facilitate effective recruitment and retention of excelling and dedicated surgeons. In 2006, Pololi discussed a 9-step ADP to support career development in academic medicine.^2^ We propose a modified 12-step ADP that can be employed by medical students, surgical residents/fellows, and junior surgeons’ faculty as they navigate potential careers in academic surgery (Supplemental Table 1, http://links.lww.com/AOSO/A90).

### Step 1: Identify a Mentor Within Academic Surgery and Outline Both the Mentor and Mentee’S Expectations

Supportive mentors who can provide guidance and feedback are a critical part to any career; however, a lack of effective mentoring is still observed among faculty members, residents, and medical students, especially among women and underrepresented minorities.^2,3^ The significance of mentorship has been repeatedly demonstrated^4,5^ and meaningful mentor-mentee interactions have been associated with increased scholarly productivity, improved professional outlook, and satisfaction in future careers.^5,6^ One study investigating the impact of formal mentorship programs on the professional and academic success of otolaryngology surgery residents demonstrated that implementation of mentorship resulted in significant improvements in the residents’ emotional scores (*P* < 0.0001), sense of personal achievement (*P* < 0.0001), levels of depersonalization (*P* < 0.0001), and quality of life (*P* = 0.003).^7^

Mentors should ideally be a faculty member in academic surgery who can (1) allocate time and effort to mentorship, (2) provide research and training opportunities, (3) offer constructive feedback, and (4) support networking. An outstanding mentor (1) provides a vision but with purposeful tailoring the vision to each mentee, (2) exhibits admirable personal qualities, including compassion and enthusiasm, (3) acts as a career guide with regular provision of feedback and meaningful suggestions, (4) respects for personal privacy and mentees’ academic-personal balance, and (5) is willing to commit time and effort toward regular and high-quality meetings.^8,9^ To successfully identify a mentor, consider your specific goals and your expectations from the mentor (i.e., research opportunities, clinical training, teaching, general advice), and short-list faculty members who you feel may be most compatible. Request the potential mentors to express their expectations of the student and take that into consideration when making your decision. The values and expectations of both the mentee and mentor should be mutually identified, thereby mitigating the risk of mentor-mentee mismatch. The success of the mentor-mentee relationship is largely reliant on the proper and early alignment of these expectations. Mentoring contracts and signed agreements to clarify these expectations are widely regarded as useful tools that facilitate open communication and understanding between the mentor and mentee.^10^

Increasing time constraints, expectations for students to initiate mentorship, and scheduling conflicts limiting in-person visits are some of many impediments to effective mentoring, and Santiesteban et al discusses potential solutions to mitigate these issues.^5^ Usage of video-conferencing and digital platforms facilitates ease of accessibility, address time constraints, and allow students to identify mentors who may be in different geographic regions. Additionally, institutionally established mentorship programs, faculty mentorship directory, and meet and greet events could serve as initiatives for mentor identification, thus easing the burden on the student alone.^5^

Additionally, the ADP should be viewed as a joint document between the mentee and mentor.

### Step 2: Define and Prioritize Your Values and Interests

An individual’s values and interests are greatly influential in the development of personal and career goals and thus should influence their ADP. Values such as honesty, family duties, intellectual challenge, physical and mental well-being, financial status, compassion, empowerment, and work-life balance shape one’s vision for their future.^11^ To develop an ADP for a growing career in academic surgery, we must first demonstrate introspection and consider what governing values and interests influence our aspirations. Through a reflective exercise, medical professionals can improve their self-understanding of their own values, interests, and strengths, as well as how these influence their careers. We recommend that you reflect upon and write a list of what you value most with a brief reasoning statement as well as their relative priority. Deliberation of personal interests, relationships with family, friends and colleagues, current and prior work experiences, and professional commitments can assist with the prioritization.^2^ Thorough contemplation of one’s values and a comprehensive list fundamental to the completion of an effective ADP, so we recommend that you spend a considerable amount of time on this step.

### Step 3: Perform a Detailed Self-assessment With Critical Appraisal of Areas of Strengths and Weaknesses

Identification of both strengths and weaknesses are key to molding future goals and the ADP.

When considering strengths, reflect on academic successes and what made them successful. Appraisal of weaknesses should motivate you to further develop skills and knowledge within those areas, shaping future goals and objectives. In this step, we recommend making separate lists for strengths and weaknesses. For each input, note why this is a strength or weakness with a clarifying statement.

### Step 4: Consider the Roles and Responsibilities of Professionals in Academic Surgery

Prospective academic surgeons should pursue their careers with strong understandings of the nature of potential jobs. Often times, a lack of understanding of career options and the professional requirements within academia can demotivate individuals and limit the recruitment and retention of future generations of professionals within this field.^11^ Understanding of these professional requirements and their challenging nature should not be a deterrent but rather a prompt for preemptive preparation to tackle these demands.

First, attempt to define academic surgery roles and responsibilities on your own. Then, involve your mentor to discuss your perception of academic surgery and request they provide their insight into the field. Guilliames et al outlined the impact of filling the gap of medical students’ and residents’ knowledge regarding careers. The authors of this study demonstrated that workshops, conferences, meaningful discussions, and other efforts to promote awareness of careers in academia among individuals can be instrumental in improving recruitment of individuals.^12^ Therefore, workshops, conferences, and personal discussions with mentors are all valuable ways to gain insight into academic surgery.

### Step 5: Identify Views and Goals Regarding and Pursue Experience in the Following Themes: (1) Research and Scholarship, (2) Academic Recognition, (3) Educational Opportunities, and (4) Teaching Activities

1. **Research and Scholarship:** Research involvement constitutes (1) procurement of funding via grants, (2) identification of research opportunities and mentors, (3) submission of manuscripts to journals, and (4) attendance at conferences.^13^ Numerous organizations and institutions offer scholarships and funding opportunities for medical professionals, some of which are outlined by Sanfey et al.^13^ One study assessing the impact of a research scholarship among female medical students, surgery residents, and fellows interested in cardiothoracic surgery research demonstrated that receipt of this scholarship was associated with successful achievement of career milestones in cardiothoracic surgery at significant rates.^14^ Early involvement in research and receipt of grant funding could advance your career in academic surgery, and we encourage you to explore opportunities with assistance from your mentor.
2. **Academic Recognition:** Networking with others in academic surgery is crucial for those entering the profession, especially through national conferences and meetings, participation in surgical societies, and involvement in surgery-oriented organizations.^15^ Opportunities to speak as session speaker at a conference, serve as a member of a meeting organization committee, or present advancements in research are highly regarded and pave the way to establishing oneself as a valuable contributor to the field of academic surgery. Furthermore, these extracurricular endeavors offer individuals the chance to receive certifications, continued medical education credits, new learning experiences, and the opportunity to network.^15^
3. **Educational Opportunities:** At every level, from medical student to faculty member, individuals should seek out opportunities to deepen their knowledge through workshops, shadowing, research, mentorship, conferences, and more. At the level of medical students, additional exposure to surgical training can be obtained via suturing and anatomy workshops, surgeon-directed formal lectures on relevant topics and skills, small-group interactive didactic sessions, and third- and fourth-year surgical clerkships. Progression to the role of resident physician provides more immersive opportunities, and we suggest residents seek opportunities in educational research, regional and national conferences, mentorship, and skills laboratories to further strengthen the skills sought in an academic surgeon. Early-career faculty members are encouraged to take similar steps as the ones previously mentioned for residents, but they should place more focus on developing their identity as a surgeon-educator, mentor, and surgeon-scientist.^13^
4. **Teaching Activities:** Individuals should seek out teaching experiences and accomplishments, which can be further categorized as (1) teaching, (2) curriculum development, (3) mentoring, and (4) educational leadership. As an aspiring academic surgeon, you should search for opportunities in one or more of these categories and document them in a teaching portfolio (the University of Virginia’s Academy of Distinguished Educators’ template is one example).^13,16,17^

### Step 6: Leverage Social Media

Social media platforms, including Twitter, Facebook, and LinkedIn, have been largely adopted by medical faculty and physicians-in-training for education and advocacy purposes.^18^ These platforms can provide a unique avenue for mentorship and outreach opportunities, especially to those who find mentorship and networking opportunities at their own institutions lacking. The use of free and online communication platforms enables inclusivity, engages intellectual conversations without the limitations of time or distance, promotes collaborations among surgeons and different specialties, and provides opportunities for mentorship and scholarship.^19^ Other social media platforms such as LinkedIn, Facebook, and Instagram have also be used with similar benefits.^20^ We encourage you to engage in these social media communities and make use of the opportunities presented to you on these platforms.

The primary danger of social media for rising medical professionals is the public availability of personal information. Although a violation of privacy, public access to private photographs, videos, and texts puts individuals’ reputations at risk. One study investigated the social media presence of 996 surgical residents from 57 residency programs and identified that 32% of these residents had identifiable Facebook profiles. The authors noted that 14.1% of these residents’ profiles displayed potentially unprofessional content and 12.2% displayed clearly unprofessional content, which were defined as Health Insurance Portability and Accountability Act violations, suggestive and explicit photos, and binge drinking.^21^ The ease with which these researchers accessed the residents’ profiles highlights the risks associated with having an active social media profile. Therefore, we caution you not to publicly post any content that may be deemed unprofessional.

### Step 7: Outline Career/academic Objectives for Each of the Next 5 Years

Rising academic surgeons are faced with a myriad of clinical and academic opportunities and interests, and they may face uncertainty regarding which interests will actually be sustainable throughout their careers.^22^ To develop further clarity, we prompt you to contemplate on what you want your professional identity to be beyond surgeon. Professional identities can include educator, innovator, leader, advocate, or scientist, and you can take on multiple roles to fit your goals. Attending faculty development and education-focused meetings and having open discussions with peers and faculty pursuing varying interests is one way to familiarize yourself with the various routes within academic surgery and determine which career goals feel like a good fit.^22^ Actively seeking out opportunities to explore your careers will assist you in identifying career goals that align best with your values and interests. Ask yourself the following questions to further assist you:

- What do I want to accomplish within the next year? What do I want to accomplish over the next 5 years?
- What are my current responsibilities at work, and what do I want my responsibilities to be?^22^
- Do my personal career goals align with the work responsibilities?^22^ If not, how can I alter my career trajectory to ensure that they align?

Once you have an understanding of your answers to these questions, develop annual goals for the next 5 years and give special consideration to how these goals fit with previously stated values in step 2. As you proceed through your career, keep in mind that regular reassessment of career goals is encouraged and may allow you to focus on your priorities and redirect your efforts toward new or revised goals.^22^

### Step 8: Outline Skills, Activities, or Steps Needed to Achieve These Goals

Break down your objectives into a list of skills and activities necessary for their achievement, which can help with personal motivation and accountability. The primary goal of this step is to devise a meaningful short-term plan with a “to-do” list of actionable items. These items may involve regular meetings with your mentor to discuss your progress, development of your skillset in a particular area of medicine, and participation in research projects and conferences, community outreach and volunteer services, student advocacy movements, and teaching experiences. Construction of a timeline or calendar to display these objectives, activities, actionable items would be an effective organizational strategy to monitor your progress and obtain your set goals.^22^

### Step 9: Determine Long-term (>10 year) Goals

Defining personal career and academic goals can be challenging for residents and junior faculty. In step 7, we prompted you to consider your annual and short-term goals. It is equally important to identify your long-term goals as well. We encourage you to consider your professional identity once again in this step. Professional identities can include educator, innovator, leader, advocate, or scientist, and you can take on multiple roles to fit your goals. Continue to explore the multifaceted nature of the academic surgical field, discuss with faculty members, and devise a plan based on your short-term goals outlined previously.^22^ Use your responses to the prior steps and ask yourself the following questions as you write out your long-term goals:

- Where do I ultimately see myself in 10 years?
- What professional identities do I want to identify with?
- How do I envision my personal life and work-life balance?
- What areas within medicine (and academic medicine) do I want to focus on particularly?

Assess if these goals are consistent with your governing values and interests and whether your short-term goals support your long-term objectives.

### Step 10: Design a Learning Contract for Each of the Objectives and Tasks

Develop a learning contract that will provide a structured documentation outlining your individualized approach to achieving your goals. The learning contract can be viewed as an efficient tool to facilitate mentee-centered activities and improve sense of personal responsibility.^23^ Completion of written document clearly stating your goals will likely increase the sense of responsibility and commitment to the efforts.^24^ A previous study investigating the impact of learning contracts on self-directed development and satisfaction among nursing students demonstrated that students with a well-developed learning contract seemed to have significant improvements in their overall satisfaction and self-directed learning tasks (*P* = 0.019).^23^ We believe that development of learning contracts by medical students, resident physicians, and junior faculty focused on the goals discussed with their mentor would produce similar results. Utilization of a learning contract was previously outlined in Pololi’s strategy, and we agree this is beneficial to the individual’s goals, commitment, and personal accountability.^2^ The learning contract should be reviewed with your mentor and should be revisited in regular intervals to assess progress.

### Step 11: Consider Potential Barriers You May Face

During the advancement from medical school to residency and junior faculty, many individuals face barriers that can hinder their progression and success in academic surgery. These barriers may come in the form of improper work-life balance, medical conditions, toxic competitive hierarchies, financial disadvantages, and lack of diversity and inclusion or even outright discrimination at a given institution.^25–27^ Anticipation of these potential barriers and planning for ways to navigate them are instrumental to success within academic surgery. To focus on the last point, there continue to be significant barriers to women, underrepresented racial minorities, and LGBTQ+ people in surgery. Women are still a minority in academic surgery, holding only 40% of surgery residency positions.^28^ Women in surgical training also suffer higher attrition rates compared to men (24% vs 16%, *P* < 0.001).^29^ Additionally, women are less likely to receive promotions to ranked faculty positions.^28^

Underrepresentation of women extends to national conferences and other areas representative of honor and acceptance by the academic surgery community. In one retrospective analysis of gender trends among speakers at national trauma surgery conferences between 2016 and 2019, the proportion of women surgeon speakers significantly increased in some major trauma associations’ annual meetings (AAST: 27.1% vs 41.4%, *P* < 0.00001; EAST: 25.2% vs 39.8%, *P* = 0.049) and did not show much difference in others (WTA: 27.8% vs 33.3%, *P* = 0.573).^30^ This study simultaneously demonstrates a cause for celebration given the improving proportions while also highlighting the need for further recruitment and invitation of women speakers given the inconsistent results among different organizations. Another study noted the proportion of women speakers had a direct correlation with the number of women in the organizing committees of the events (r = 0.71, *P* < 0.001).^31^ Therefore, increased recruitment of women into organizational leadership positions could be a potential avenue to increase representation of women not only in national conferences but also within higher ranking faculty and administrative positions.^32^

Racial disparities in academic surgery are also still present to this day. Black assistant professors in academic surgery are reported to have lower 10-year promotion rates compared to white faculty (*P* < 0.01),^33^ and minority faculty (Asian, Black, or Hispanic) have lower retention ratees compared with white faculty (*P* < 0.01).^33^ Additionally, LGBTQ+ people face unique stressors in training, with one study reporting that nearly 57% of LGBTQ+ surgical residents concealed their sexual orientation due to fears of poor evaluations and mistreatment.^34^

We encourage you to take the time to process the information and statistics provided in this step to contemplate what potential barriers you foresee in your own careers. Please write down these potential barriers and work with your mentor to develop plans to navigate through these situations in the future. We suggest that you continue to have open dialogue regarding these issues with your mentors and institutional faculty to enhance your self-awareness. Regardless of whether you identify with any of these categories, we encourage you to seek opportunities to understand the challenges faced by members of these groups and discuss how you can address them in your career. We suggest you participate in women-, LGBTQ-, or racial diversity-centered collaborative efforts as well as attend conferences and workshops on diversity, equity, and inclusion (DEI) in academic surgery. We also strongly encourage all institutions to proactively work to end disparities based on gender, race, and sexual orientation through robust DEI initiatives as well as by hiring and promoting more individuals from these groups, especially to senior/leadership positions.

### Step 12: Plan Evaluations of Progress and Feedback at Regular Intervals

Evaluation of progress is a critical element for an effective ADP and can foster support and accountability. Assessment in regular intervals would be recommended since this can foster a sense of support and accountability.^35,36^ One potential strategy is to hold regular meetings with your mentor to revisit goals and associated tasks to assess if they have been achieved, are in progress, or have been unaddressed. The method of evaluation should be tailored to the preferences of the mentee and mentor.^35^ Meaningful feedback from faculty mentors and supervisors is strongly encouraged to facilitate student and resident development. However, faculty often are limited in time or formal assessment training, thus lowering the impact that the feedback can have on students. Therefore, we encourage more emphasis on time allocation for feedback sessions and formal assessment training for all mentors.^36^

## CONCLUSIONS

The role of an academic surgeon can be defined as one who serves as an innovator, educator, and expert in addressing complex clinical problems while disseminating knowledge and providing training for future generations of surgeon scientists.^37^ Future academic surgeons should understand the specifics of the career they are pursuing. The proposed 12 steps have the potential to serve as the initial building blocks for a rising surgeon’s development of emotional intelligence, a critical trait among surgeons. In fact, training programs are placing increased emphasis on emotional intelligence cultivation as the resident progresses through training via workshops, didactics, and mentorship.^38^ Surgeon mentors should support the recruitment and retainment of academic surgeons through effective academic career support and strategic planning. Intentional career development can serve as an essential tool as both mentors and mentee navigate different career choices, and we strongly encourage all to consider completing their own ADP as a significant step toward achieving a successful academic career advancement.
